# Eicosanoids in platelets and the effect of their modulation by aspirin in the cardiovascular system (and beyond)

**DOI:** 10.1111/bph.14196

**Published:** 2018-04-19

**Authors:** Marilena Crescente, Laura Menke, Melissa V Chan, Paul C Armstrong, Timothy D Warner

**Affiliations:** ^1^ Centre for Immunobiology, Blizard Institute, Barts and The London School of Medicine and Dentistry Queen Mary University of London London UK

## Abstract

Platelets are important players in thrombosis and haemostasis with their function being modulated by mediators in the blood and the vascular wall. Among these, eicosanoids can both stimulate and inhibit platelet reactivity. Platelet Cyclooxygenase (COX)‐1‐generated Thromboxane (TX)A_2_ is the primary prostanoid that stimulates platelet aggregation; its action is counter‐balanced by prostacyclin, a product of vascular COX. Prostaglandin (PG)D_2_, PGE_2_ and 12‐hydroxyeicosatraenoic acid (HETE), or 15‐HETE, are other prostanoid modulators of platelet activity, but some also play a role in carcinogenesis. Aspirin permanently inhibits platelet COX‐1, underlying its anti‐thrombotic and anti‐cancer action. While the use of aspirin as an anti‐cancer drug is increasingly encouraged, its continued use in addition to P_2_Y_12_ receptor antagonists for the treatment of cardiovascular diseases is currently debated. Aspirin not only suppresses TXA_2_ but also prevents the synthesis of both known and unknown antiplatelet eicosanoid pathways, potentially lessening the efficacy of dual antiplatelet therapies.

**Linked Articles:**

This article is part of a themed section on Eicosanoids 35 years from the 1982 Nobel: where are we now? To view the other articles in this section visit http://onlinelibrary.wiley.com/doi/10.1111/bph.v176.8/issuetoc

**Abbreviations**
AA: arachidonic acid
CYP450: cytochrome P450
EETs: epoxyeicosatrienoic acids
ECs: endothelial cells
HETE: hydroxyeicosatraenoic acid
LOX: lipoxygenase
NSAIDs: nonsteroidal anti‐inflammatory drugs
PGI_2_: prostacyclin
PUFAs: polyunsaturated fatty acids
USPSTF: US Preventive Services Task Force

## Introduction

Platelets play a fundamental role in maintaining haemostasis. A fine balance exists in which platelets can be rapidly activated to aggregate and form a plug that prevents bleeding. But when platelets get inappropriately activated, thrombi form within the vessel wall which can lead to thrombotic events such as heart attack and stroke. The activation or inhibition of platelets can be modulated by many agents with a central role being played by eicosanoids. TXA_2_ and prostacyclin (PGI_2_) are the main eicosanoids affecting the function of platelets. The groups of Vane and Samuelsson were pioneers in their identification and in establishing their action on platelets and on the vasculature (Bunting *et al.,* 1977; Bunting *et al.,* 1983; Moncada *et al.,* 1976; Moncada *et al.,* 1978; Needleman *et al.,* 1976; Svensson *et al.,* 1975; Whittaker *et al.,* 1976).

Since their discovery, and with the continued development of analytical techniques such as mass spectrometry‐based lipidomics, hundreds of structurally and stereochemically distinct eicosanoid families have been identified (Harkewicz and Dennis, 2011).

This review will focus on the production of eicosanoids by platelets and endothelium and their effect on platelet function in the cardiovascular system. We will discuss how aspirin modulates the synthesis of these eicosanoids and the consequences on its anti‐thrombotic efficacy. Laboratory techniques to evaluate response to aspirin will be also presented, and their ability to predict the occurrence of cardiovascular events will be examined. Finally, recent advances in understanding the role of platelet‐related eicosanoids in cancer will be presented.

## Eicosanoids and the fine regulation of platelet function and haemostasis

Eicosanoids are mainly derived from arachidonic acid (AA) but can also be generated from other 20 carbon polyunsaturated fatty acids (PUFAs), such as dihomo‐γ‐linolenic acid, an ω‐6‐derived PUFA, or eicosapentaenoic acid (Subhash *et al.,* 2007). These fatty acids are released from the cellular phospholipid membrane *via* the action of the enzyme phospholipase A_2_ (PLA_2_) and subsequently converted *via* the COXs into TXA_2_ and PGs, such as PGI_2_, PGE_2_ and PGD_2_, *via* lipoxygenases (LOXs) into hydroxyeicosatraenoic acids (e.g. 12‐HETE), and *via* cytochrome P450 (CYP450) enzymes into epoxyeicosatrienoic acids (EETs) (Dennis and Norris, 2015)**.**

**Figure 1:**
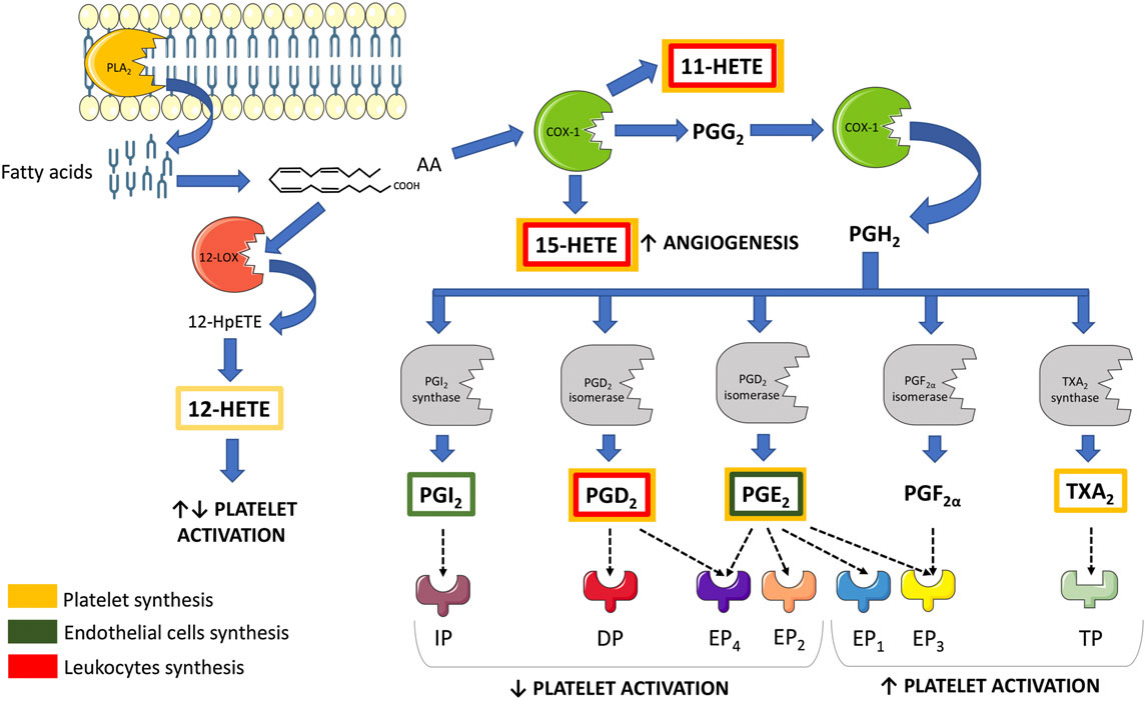
Diagram of the biosynthesis of the main eicosanoids that affect platelet function and where it occurs. The yellow, green and red boxes represent the origin of the eicosanoids as platelets, ECs and leukocytes respectively. The receptors for each eicosanoid are shown as well as the associated effects on platelet activation.

Platelets can produce significant amounts of TXA_2,_ PGE_2_, PGD_2_, 11‐, 12‐ and 15‐HETE dependent upon the activity of cytosolic group IV A PLA_2_, a widely expressed PLA_2_ isoform (Kirkby *et al.,* 2015; Rauzi *et al.,* 2016). Below, we will discuss platelet and non‐platelet‐derived eicosanoids whose actions modulate platelet function and consequentially haemostasis and thrombosis (Figure 1).

### COX‐dependent eicosanoids

COX, more precisely known as PGH synthase, converts AA first into PGG_2_, *via* a COX function and then to PGH_2_ following a peroxidase reaction (Smith and Dewitt, 1996). PGH_2_ is an unstable molecule and, in platelets, undergoes further transformations catalysed by TX synthase, PGD isomerase or PGE synthase to form TXA_2_, PGD_2_ or PGE_2_ respectively.

Two different isoforms of COX exist in the cardiovascular system, namely, COX‐1 and COX‐2 (Hla and Neilson, 1992; Kujubu *et al.,* 1991; Masferrer *et al.,* 1992; O'Banion *et al.,* 1992; Xie *et al.,* 1991). COX‐1 is usually considered a constitutive form (Kirkby *et al.,* 2012; Langenbach *et al.,* 1997), while COX‐2 is considered to be an inducible enzyme, although a role for constitutive COX‐2 has been shown in the kidneys and the central nervous system (Herschman *et al.,* 1997; Mitchell and Warner, 2006). Platelets mainly express COX‐1, but traces of COX‐2 have been detected, possibly carried over from megakaryocytes, the platelet precursor cells, or as a result of the transcription of residual mRNA into protein (Rocca *et al.,* 2002; Warner *et al.,* 2011).

### Thromboxane A_2_

The most directly important prostanoid for platelet function is COX‐1‐generated TXA_2_. It was first identified by Vane as a ‘rabbit‐aorta‐contracting substance’ (RCS) produced by the lungs during anaphylaxis (Piper and Vane, 1969). Later, TXA_2_ was shown to be synthesized by activated platelets and to act in an autocrine and paracrine manner to induce thrombosis (Smith and Willis, 1971). On platelets, TXA_2_ binds to the thromboxane prostanoid (TP) receptor and initiates an amplification loop leading to further platelet activation, aggregation and TXA_2_ formation (Reilly and Fitzgerald, 1993). The TP receptor can couple with several G proteins, such as G_12/13_, leading to platelet shape change *via* phosphorylation of the myosin light chain, platelet granule release and irreversible aggregation (Smyth, 2010). In the vasculature, TXA_2_ induces vasoconstriction and the proliferation of vascular smooth muscle cells.

### PGI_2_ (prostacyclin)

When first discovered as an autacoid produced by vascular tissue, PGI_2_ or prostacyclin was named as PGX and was described as a substance which, in contrast to TXA_2_, inhibited the clumping of platelets and relaxed vascular strips (Moncada *et al.,* 1976). Now known to be predominantly produced by the endothelium within blood vessels, there has been strong debate as to which isoform of COX catalyses the vascular production of PGI_2_. Although still controversial, research by ourselves and colleagues strongly suggests that, in the healthy vasculature, PGI_2_ production is driven by COX‐1 (Bolego *et al.,* 2009; Evangelista *et al.,* 2006; Kirkby *et al.,* 2012; Yu *et al.,* 2012). This is discussed in more detail elsewhere in this issue (Mitchell and Kirkby, 2018).

Endothelium‐produced PGI_2_ binds to the G_s_‐coupled PGI_2_ receptor (IP) on platelets and generally reduces platelet reactivity, which can be critical to minimizing the risk for atherothrombotic events (Midgett *et al.,* 2011). Binding of PGI_2_ to the IP receptor results in the activation of adenylate cyclase and a subsequent rise in cAMP levels in platelets (Yang *et al.,* 2002). This stimulates phosphorylation of PKA, which suppresses various signalling pathways involved in platelet function such as adhesion, aggregation and granule secretion. With regard to the subject of this review, PKA activation decreases the release of Ca^2+^ from internal stores, reducing the activation of cytosolic PLA2 (cPLA_2_) and the liberation of AA from the phospholipid membrane, and so diminishing the production of platelet‐derived eicosanoids, such as TXA_2_ (den Dekker *et al.,* 2002).

### PGD_2_

PGD_2_ is well established as a macrophage product but, in lesser amounts, is also synthesized by platelets. By interaction with platelet DP_1_ receptors, PGD_2_ increases adenylyl cyclase activity and so, like PGI_2_, inhibits platelet activation (Bushfield *et al.,* 1985; Oelz *et al.,* 1977; Whittle *et al.,* 1978).

### PGE_2_

PGE_2_ is released by endothelial cells (ECs) and, to some extent, by activated platelets. It acts on a range of prostanoid receptors, EP_1_ ‐ EP_4_, that differently modulate second messengers, such as cAMP and free Ca^2+^, within platelets and exert contrasting effects on platelet function (Deeb *et al.,* 2008; Yang *et al.,* 2002). The effects on platelets of PGE_2_ acting through EP receptors are concentration dependent. At low concentrations (0.1–10 μmol·L^−1^), PGE_2_ binds to G_i_‐coupled receptors (EP_3_) to enhance aggregation, whereas at higher concentrations (100 μmol·L^−1^), it activates G_s_‐coupled receptors (EP_2_, EP_4_) to inhibit aggregation (Friedman *et al.,* 2015; Glenn *et al.,* 2012; Petrucci *et al.,* 2011). Stimulation of EP_3_ receptors by PGE_2_ decreases cAMP levels, thus favouring platelet aggregation, but the full effect is only seen in the presence of another platelet agonist (Fabre *et al.,* 2001; Friedman *et al.,* 2015). On the other hand, the increased cAMP levels which accompany EP_4_ receptor activation correlate with suppressed platelet aggregation (Glenn *et al.,* 2012).

In addition to PGE_2_, PGE_1_, PGF_2α_ and PGD_2_ can also bind to EP_3_ and EP_4_ receptors but with lower affinity and reversible effects (Armstrong *et al.,* 1985; Friedman *et al.,* 2015; Glenn *et al.,* 2012).

As well as the well‐characterized effects of PGE_2_ mediated through EP_3_ and EP_4_ receptors, EP_1_ receptors are also expressed on platelets (Kauskot and Hoylaerts, 2012; Petrucci *et al.,* 2011). Although the signal transduction pathway is not clear, studies in several cell lines expressing EP_1_ receptors suggest that its activation increases Ca^2+^ influx and might thereby stimulate platelet aggregation (Whittle *et al.,* 2012).

While PGE_2_ seems to both inhibit and potentiate platelet aggregation *in vitro*, a study by Gross *et al*. has elegantly shown that, *in vivo,* PGE_2_ is produced by the vessel wall or after the rupture of a plaque. Under these conditions, PGE_2_ activates the EP_3_ receptors on platelets and clearly enhances, rather than reduces, thrombus formation in the arterial vessel wall (Gross *et al.,* 2007).

### LOX‐dependent 12‐HETE

12‐HETE is the major 12‐LOX‐catalysed metabolite and the most abundant eicosanoid produced by platelets upon stimulation (Kirkby *et al.,* 2015; Rauzi *et al.,* 2016), but its effects on platelet function are not completely understood. Initial studies suggested that both 12‐HETE and 14‐hydroxy‐docosahexaenoic acid (14‐OH‐DHA), the 12‐LOX‐derived metabolite of DHA, inhibit platelet aggregation initiated by the TP receptor agonist U46619 (Croset *et al.,* 1988). In agreement with these data, platelet‐specific knockout of 12‐LOX in mice resulted in hypersensitivity to ADP‐induced aggregation, which was reversed by incubation with exogenous 12‐HETE. However, lack of 12‐LOX did not affect collagen‐induced aggregation or platelet adhesion (Johnson *et al.,* 1998). Interestingly, another study reported that inhibition of 12‐LOX led to decreased platelet aggregation that correlated with a significant reduction of 12‐HETE in response to collagen (Maskrey *et al.,* 2014). A recent review concluded that 12‐HETE can exert both pro‐ and anti‐aggregatory effects on platelets that depend crucially on 12‐HETE concentration, stereospecificity and co‐incubation with different agonists (Porro *et al.,* 2014). Platelets also produce hepoxilins from the precursor 12‐hydroperoxyeicosatetraenoic acid. Hepoxilin has shown to exert anti‐thrombotic effects in platelets (Margalit *et al.,* 1995), most likely *via* inhibition of TXA_2_ formation and blockade of the TP receptor (Reynaud, 2002).

## Platelet‐cellular crosstalk and eicosanoid biosynthesis

**Figure 2:**
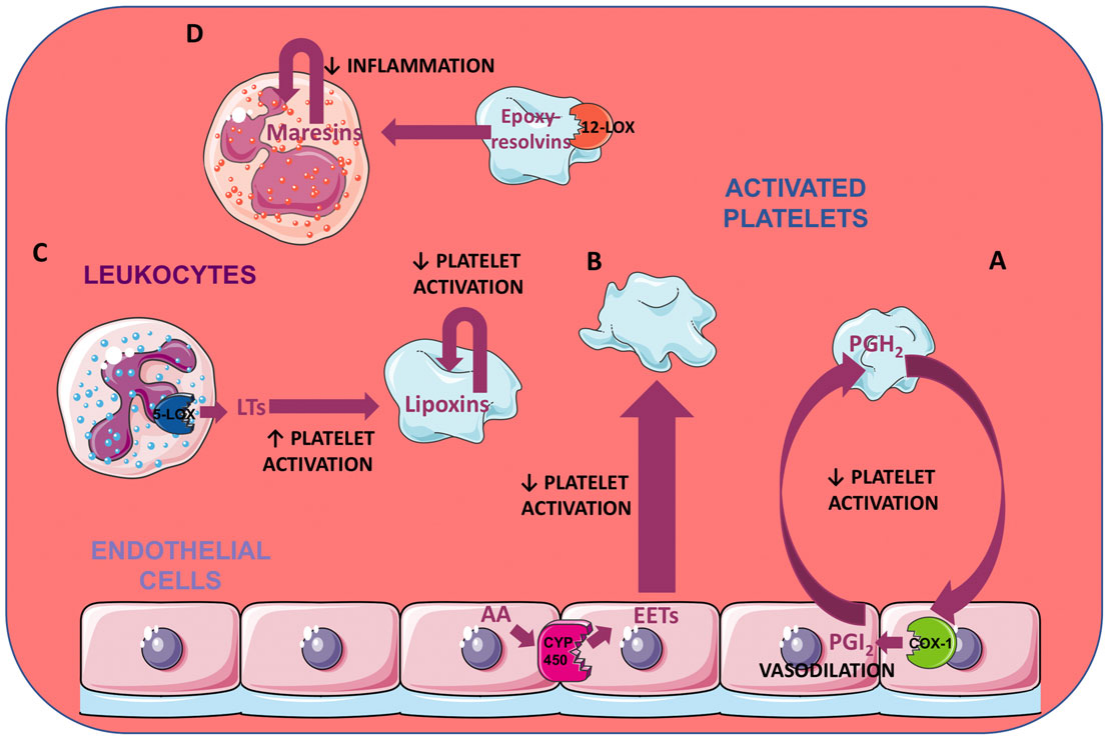
Main pathways of eicosanoid‐mediated crosstalk between platelets and other cells. The eicosanoid exchanges between platelets and ECs and their effects on the vessel homeostasis are illustrated in (A) and (B). Some of the PGH_2_ released by platelets may be used by COX‐1 in the ECs to produce PGI_2_ which induces vasodilation and prevents further platelet activation (A). ECs, on the other hand, can synthesize EETs starting from AA, through the action of CYP450. EETs reduce platelet activation (B). (C) and (D) represent some routes of platelet‐leukocyte crosstalk. LTs are synthesized in leukocytes by 5‐LOX and act together with other agonists to potentiate platelet activation. However, platelets can also use LTs to make lipoxins which reduce the activation of platelets (C). 12‐LOX in platelets also produces epoxy‐resolvins that can be used by the leukocytes to make maresins, molecules important for the resolution of inflammation (D).

Transcellular routes through which platelets exchange eicosanoids with ECs or leukocytes are important to vascular homeostasis as well as to processes such as vascular inflammation. Some of these cellular crosstalk pathways are depicted in Figure 2 and discussed below. For example, ECs can utilize PGH_2_ released from platelets to produce PGI_2_. This suggests a counteractive mechanism in which activated platelets that are in direct contact with the vessel wall produce endoperoxide that can in turn be used by ECs to inhibit platelet functions and stimulate the return to homeostasis (Marcus *et al.,* 1980; Porro *et al.,* 2014).

CYP450 epoxygenases can convert AA into the biologically active EETs. The main producers of EETs are vascular ECs which not only release EETs following stimulation and contribute to vasodilation but also promote anti‐inflammatory effect in the vascular system (Yang, 2015). EETs also have potent anti‐adhesive and anti‐aggregatory activities which they exert by causing hyperpolarization of the platelet membrane (Sudhahar *et al.,* 2010).

In the cardiovascular system, leukocytes represent the main source of 5‐LOX‐derived LTs. These metabolites potentiate adrenaline and thrombin‐induced platelet aggregation, probably by increasing the activity of TXA_2_ synthetase and thereby TXA_2_ formation (Mehta *et al.,* 1986). On the other hand, platelets can utilize leukocyte‐derived LTA_4_ as a precursor for lipoxin production. Following release, lipoxin A_4_ acts on platelets *via* the FPR2/ALX receptor (Czapiga *et al.,* 2005) and mediates protective functions by suppressing platelet adhesion, TXA_2_ formation and platelet–neutrophil interaction (Ortiz‐Muñoz *et al.,* 2014). With regard to inflammation, platelets can transfer eicosanoid precursors to leukocytes which are fundamental for the formation of pro‐resolving mediators. A prominent example is the epoxy‐resolvins, which are produced by platelet 12‐LOX and transferred to neutrophils where they are transformed into maresins, which are molecules with important roles in terminating acute inflammatory responses (Abdulnour *et al.,* 2014).

## Modulation of eicosanoid production by platelets and the anti‐thrombotic efficacy of aspirin

John Vane reported for the first time that aspirin inhibits the production of PGs (Vane, 1971). This mechanism was identified as the basis of the therapeutic action of nonsteroidal anti‐inflammatory drugs (NSAIDs) (Vane, 1971) and was confirmed in platelets by Smith and Willis (1971). Many NSAIDs have been developed since then, and we know now that these compounds affect eicosanoid biosynthesis through the inhibition of both COX‐1 and COX‐2. COX‐1 and COX‐2 are expressed to differing levels in different tissues and under different conditions of health and disease. Such differences and their significance has been reviewed extensively (Khan *et al.,* 2002; Mitchell and Warner, 2006; Wallace and Devchand, 2005).

In the context of platelet function, only aspirin produces irreversible inhibition of COX‐1 through its ability to covalently modify the enzyme (Cerletti *et al.,* 1982; Loll *et al.,* 1995). Consequently, aspirin impairs the synthesis of TXA_2_ for the entire platelet lifespan, and this explains its general antithrombotic action (Ferreira *et al.,* 1971; Smith and Willis, 1971; Vane, 1971), although under some circumstances aspirin‐treated platelets may be able to recover the ability to synthesize TXA_2_ after *de novo* synthesis of COX‐1 (Evangelista *et al.,* 2006). Because of its irreversible action, the antiplatelet effects of aspirin are seen with low doses of 50–100 mg·day^−1^ (Patrignani *et al.,* 1982; Patrono, 2005; Warner *et al.,* 2011). Aspirin is commonly given in combination with antagonists of ADP, acting at P_2_Y_12_ receptor, such as clopidogrel, prasugrel or ticagrelor (Bhatt, 2009; Gargiulo *et al.,* 2016; Investigators TCIUaTPRET, 2001; Patrono *et al.,* 2011; Wallentin *et al*., 2009; Windecker *et al.,* 2014; Wiviott *et al*., 2007). Despite the proven anti‐thrombotic efficacy of this dual therapy, many studies are currently investigating the benefits of single antiplatelet‐drug therapy, using newer drugs such as ticagrelor (Gargiulo *et al.,* 2016). The hope is to retain the anti‐thrombotic effects of dual antiplatelet therapy while lessening the unwanted side effects. This rationale is not only based on the need to reduce the bleeding risk associated with the dual antiplatelet therapy (Du *et al.,* 2016; Maree and Fitzgerald, 2007) but also because evidence suggests that P_2_Y_12_ antagonists alone can decrease platelet TXA_2_ production and reduce aggregation mediated by TP receptor activation (Armstrong *et al.,* 2010; Armstrong *et al.,* 2011; Bhavaraju *et al.,* 2010; Kirkby *et al.,* 2011). Furthermore, the ability of aspirin to reduce the production of vascular PGI_2_ directly by inhibiting COX‐1 in ECs or indirectly by inhibiting COX‐1 in other cells supplying precursors of PGI_2_, such as PGH_2_, could produce a pro‐thrombotic effect that reduces the overall efficacy of dual antiplatelet therapy (Björkman *et al.,* 2013; FitzGerald *et al.,* 1983; Franchi *et al.,* 2016; Mahaffey *et al.,* 2011; Maree and Fitzgerald, 2007; Warner *et al.,* 2010; Warner *et al.,* 2016). Therefore, it is necessary not only to seek therapeutic strategies apart from aspirin, but also to extensively re‐evaluate the effects of aspirin *in vivo*. This last goal could be achieved by using more recently developed techniques such as liquid chromatography–tandem mass spectrometry or the genetic manipulation of animals. For example, we have recently found, through the use of mass spectrometry analysis, that aspirin prevents not only the synthesis of TXA_2_ by platelets but also the production of PGD_2_, PGE_2_, 11‐HETE and 15‐HETE. PGD_2_ and PGE_2_ are PGs with antiplatelet actions and their inhibition can further contribute to a reduced efficacy of the antithrombotic treatments (Rauzi *et al.,* 2016). In addition, our own recently developed animal models where the expression of COX‐1 is specifically ablated in ECs or in megakaryocytes/platelets will be useful in dissecting the effects of eicosanoids on the cardiovascular system and the outcomes of aspirin treatment.

## Eicosanoid measurements and platelet function tests to evaluate the efficacy of aspirin in cardiovascular patients

The way platelets respond to treatment with aspirin can be monitored in the laboratory either by techniques that specifically measure platelet COX‐1 activity or by tests assessing other platelet activation pathways besides COX‐1.

The measurement of platelet‐generated eicosanoids, in particular of TXB_2_, the stable form of TXA_2_, either in serum or after *in vitro* stimulation of platelets, falls in the first category of techniques. With a strong stimulus, the levels of TXB_2_ can be taken as reflecting the maximal capacity of platelets to synthesize TXA_2_ *via* the COX‐1 pathway and this can be regarded as a sensitive measure of the response to aspirin, in the laboratory (Cattaneo, 2007; Maree and Fitzgerald, 2007; Ohmori *et al*., 2006). On the other hand, the levels of the main TXA_2_ metabolite found in urine, 11‐ dehydro TXB_2_, reflect systemic TXA_2_ generation and may not only reflect the effect of aspirin on platelet COX‐1 (Kirkby *et al.,* 2012; Kirkby *et al.,* 2015; Smith *et al.,* 2012).

Another standard test for studies of platelet inhibition by aspirin is light transmission aggregometry, which measures the ability of platelets to aggregate after being stimulated. Different stimuli can be used in this test to explore different aspects of platelet activation. AA is a substrate for COX‐1, so the aggregation response to this agonist closely reflects platelet COX‐1 activity, while ADP or collagen induces platelet aggregation through pathways that are not exclusively dependent on COX‐1 activation (Thiagarjan and Wu, 2002). Other methodologies, such as flow cytometry evaluation of markers of platelet activation and secretion or of the formation of platelet‐leukocyte aggregates, can also be used to assess platelet inhibition by aspirin. Moreover, semi‐automated point‐of‐care platelet function assays, such as the PFA‐100® system and RPFA‐Verify‐Now Aspirin, have been introduced (Frelinger *et al.,* 2006).

The prevalence of aspirin resistance, that is, lack of effect of aspirin, reported in the literature is largely based on various non‐specific laboratory techniques and, in general, aspirin resistance is much lower when measured with COX‐1 specific methods (Gurbel *et al.,* 2007; Lordkipanidzé *et al.,* 2007).

It is generally held that aspirin should inhibit platelet TXA_2_ synthesis by at least 95% to reach a functional effect, and this assumption is mainly based on the observation that there is a non‐linear relationship between inhibition of platelet TXA_2_ synthesis and inhibition of platelet aggregation (Kidson‐Gerber *et al.,* 2010 *;* Santilli *et al.,* 2009). However, due to the technical limitations of the tests employed, platelet response to aspirin is usually evaluated using one or two agonists, often at fixed concentration that does not make it possible to properly characterize biological variations in drug response. Recently, we have developed a test using optical multichannel platelet aggregometry in a 96‐well‐plate, that can explore platelet function in response to a broad range of agonists and agonist concentrations (Chan *et al.,* 2011; Lordkipanidzé *et al.,* 2014). This test has indicated that there is a linear relationship between TXA_2_ synthesis and TXA_2_‐mediated platelet aggregation, in the presence of different levels of COX‐1 inhibition and could represent a valid alternative method of reliably identifying responders to treatment with aspirin (Armstrong *et al.,* 2008).

**Figure 3:**
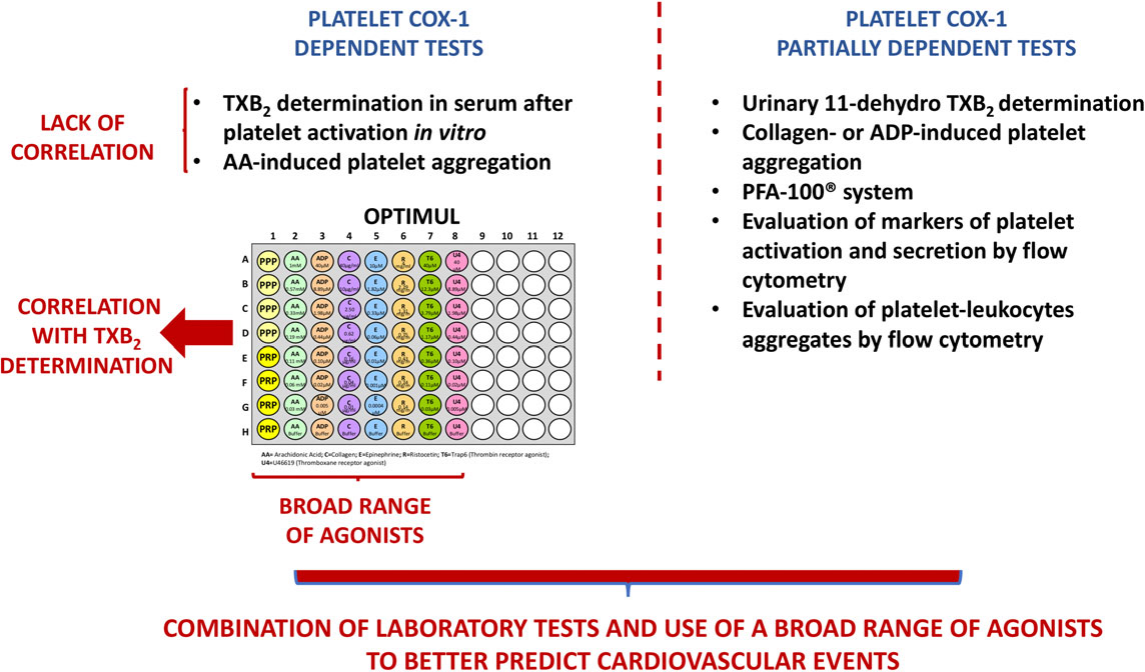
Schematic representation of platelet function tests used to monitor responses to aspirin in cardiovascular patients.

The association between a high platelet reactivity while on treatment, and the risk of patients having a thrombotic event is uncertain (Consuegra‐Sánchez *et al.,* 2013; Depta *et al.,* 2012; Li *et al.,* 2014; Tantry *et al.,* 2013). However, four different meta‐analyses have so far indicated that the lack of response to aspirin, as detected in the laboratory, may predict clinical recurrences (Crescente *et al.,* 2008a; Crescente *et al.,* 2008b; Krasopoulos *et al.,* 2008; Reny *et al.,* 2008; Snoep *et al.,* 2007). It also appears, from some of the studies performed in this area, that a combination of tests and of different agonists is better than one single test to establish this type of association (Armstrong *et al.,* 2008; Crescente *et al.,* 2011; Gremmel *et al.,* 2015; Smith *et al.,* 2012) and a summary of these observations is provided in Figure 3 **.** However, it is essential that additional biomarkers of response to aspirin are identified and larger epidemiological studies performed, before any change of an antiplatelet treatment is made on the basis of laboratory test results. Notably, there have been no clinical trials demonstrating that tailoring antiplatelet therapy to results from *ex vivo* platelet testing, produces an improvement in patient outcomes (Collet *et al.,* 2012; Depta *et al.,* 2012).

## Anti‐cancer effect of aspirin: role for platelet eicosanoids

In 1988, Kune *et al*. reported for the first time an association between the intake of aspirin and a reduced risk of colorectal cancer, thus extending the therapeutic potential of aspirin beyond its use as an anti‐inflammatory or anti‐thrombotic drug. This observation was confirmed by many subsequent epidemiological studies and by a large meta‐analysis which also showed that aspirin reduced the risk of gastrointestinal cancers in general (Algra and Rothwell, 2012; Burn *et al*., 2008; Burn *et al.,* 2011; Cole *et al.,* 2009; Cuzick *et al.,* 2015; Rothwell *et al.,* 2012). As well as aspirin, non‐aspirin NSAIDS and, in particular, COX‐2 selective inhibitors, such as celecoxib and rofecoxib, were widely reported to prevent colonic tumourigenesis (Arber *et al*., 2006; Arber *et al.,* 2011; Baron *et al.,* 2006; Bertagnolli *et al*., 2006; Cao *et al.,* 2016; Steinbach *et al*., 2000). However, concerns about the pro‐thrombotic effects of non‐aspirin NSAIDs including COX‐2 inhibitors (Baron *et al.,* 2006; Baron *et al.,* 2008; Collaboration CaTNTC, 2013) have ended cancer prevention trials using COX‐2 inhibitors , and the US Preventive Services Task Force (USPSTF) no longer supports the use of non‐aspirin NSAIDs for the prevention of colorectal cancer.

In contrast, aspirin is the only drug with no cardiovascular risk that is effective in both primary and secondary prevention of colorectal cancer and also reduces the incidence and risk of all‐cause cancer mortality (Cuzick *et al.,* 2015; Rothwell *et al.,* 2011). As aspirin is used in prevention of cardiovascular diseases and the most colorectal cancer cases are diagnosed after the age of 50, the last guidelines from the USPSTF recommend low‐dose aspirin for the primary prevention of colorectal cancer in patients at increased cardiovascular risk (Bibbins‐Domingo, 2016).

The follow‐up studies of many clinical trials indicate that the chemoprotective action of aspirin can be detected at a dose as low as 75 mg·day^−1^. Furthermore, it is saturable at these low doses and is present when using a controlled‐release aspirin formulation that mainly targets platelet COX‐1 (Patrignani and Patrono, 2016). These findings have been confirmed by studies showing that small doses of aspirin, by blocking the formation of platelet TXA_2_, PGE_2_, PG‐containing oxidized phospholipids and sphingosine 1‐phosphate, reduce the exchange of lipid mediators between platelets and cancer cells in the tumour micro‐environment (Aldrovandi *et al.,* 2013; Dovizio *et al.,* 2013; Ulrych *et al.,* 2011).

Strong evidence also suggests that eicosanoids linked to COX‐1 activity act as pro‐angiogenic factors and therefore the anti‐cancer effects of aspirin are also related to a reduction of angiogenesis (Etulain *et al.,* 2013; Rauzi *et al.,* 2016). For example, we have recently found that platelet COX‐1‐derived 15(S)‐HETE induces an angiogenic response in HMEC‐1 cells and rat aortic rings and this effect disappears in presence of aspirin, when the synthesis of 15(S)‐HETE is blocked (Rauzi *et al.,* 2016). In addition to the eicosanoids, platelets can release a variety of pro‐angiogenic factors from their α‐granules and this release can be modulated by treatment with aspirin, as well (Coppinger *et al.,* 2004).

Platelets promote cancer progression also by favouring the metastatic process. In particular, platelets will form aggregates around tumour cells in the bloodstream, that protect tumor cells from being cleared by the immune system (Gay and Felding‐Habermann, 2011). Also, when COX‐1 activity is blocked by aspirin or when a PGE_2_ antagonist is used, platelets lose the ability to transform human colon carcinoma cells into mesenchymal‐like cancer cells. Moreover, the administration of aspirin to mice prevents the platelet‐induced formation of metastases in the lungs, and this is associated with a reduced systemic synthesis of TXA_2_ and PGE_2_ (Guillem‐Llobat *et al.,* 2016).

**Figure 4:**
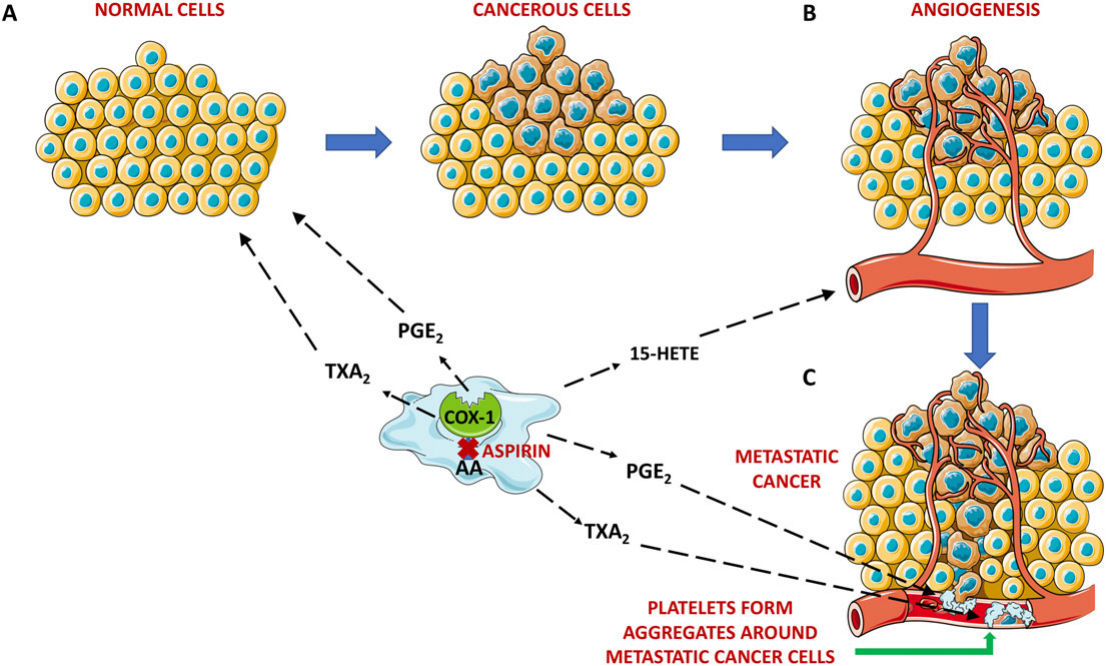
Effects of platelet COX‐1‐derived eicosanoids and of aspirin treatment in the progression of cancer. The preventive role of aspirin in the progression of cancer depends at least in part on its ability to block the formation of eicosanoids by platelet COX‐1. TXA_2_ and PGE_2_ are released in the tumour micro‐environment and favour the transformation of cells from a normal to a cancerous phenotype (A). 15‐HETE is another eicosanoid synthesised by COX‐1 in platelets that promotes angiogenesis, a process that further promotes cancer progression (B). TXA_2_ and PGE_2_ mediate the formation of platelet aggregates around the metastatic cancer cells, protecting them from the immune system and assisting their spread throughout the body (C).

This evidence suggests that the anti‐cancer efficacy of aspirin resides in its ability to block the biosynthesis of platelet‐derived eicosanoids, which not only serve as substrates for other cells present in the tumour micro‐environment but also promote angiogenesis and the metastatic progression of the tumour (Figure 4). While there is strong evidence for aspirin having beneficial effects in gastrointestinal cancers, the efficacy of aspirin in other cancer types such as gastroesophageal, breast and prostate cancers has still to be evaluated, as well as the most appropriate timings and doses that can be used to maximize its anti‐carcinogenic effects (Patrignani and Patrono, 2016).

## Conclusions

Eicosanoids produced by platelets, or made from other cells, are important modulators of platelet function and regulate the fine balance between haemostasis and thrombotic disease. The eicosanoid‐mediated crosstalk between platelets and other cells also regulates pathophysiological processes such as cancer. Low doses of aspirin, through their ability to inhibit platelet COX‐1 and the synthesis of pro‐aggregatory TXA_2_, is still nowadays considered as a first choice treatment to reduce the risk of thrombotic events. Ongoing research may lead to the replacement of aspirin in this role by P2Y_12_ receptor antagonists, while aspirin continues to be used for protection against the development of a range of cancers.

### Nomenclature of targets and ligands

Key protein targets and ligands in this article are hyperlinked to corresponding entries in http://www.guidetopharmacology.org, the common portal for data from the IUPHAR/BPS Guide to PHARMACOLOGY (Harding *et al.,* 2018), and are permanently archived in the Concise Guide to PHARMACOLOGY 2017/18 (Alexander *et al.,* 2017a,b).

## Conflict of interest

The authors declare no conflicts of interest.
